# Diagnostic and Therapeutic Potential of MicroRNAs in Lung Cancer

**DOI:** 10.3390/cancers9050049

**Published:** 2017-05-09

**Authors:** Kentaro Inamura

**Affiliations:** Division of Pathology, The Cancer Institute, Japanese Foundation for Cancer Research, 3-8-31 Ariake, Koto-ku, Tokyo 135-8550, Japan; kentaro.inamura@jfcr.or.jp; Tel.: +81-3-3570-0111 (ext. 5604); Fax: +81-3-3570-0558

**Keywords:** adenocarcinoma, asbestos, driver mutation, genetic alteration, histology, miRNA, molecular pathology, oncology, smoking

## Abstract

Lung cancer is the leading cause of deaths resulting from cancer owing to late diagnosis and limited treatment intervention. MicroRNAs are short, non-coding RNA molecules that regulate gene expression post-transcriptionally by translational repression or target messenger RNA degradation. Accumulating evidence suggests various roles for microRNAs, including development and progression of lung cancers. Because microRNAs are degraded to a much lesser extent in formalin-fixed paraffin-embedded specimens and are present not only in tumor tissues but also in body fluids, there is an increased potential in microRNA analyses for cancer research. In this review, recent studies of microRNA are introduced and briefly summarized, with a focus on the association of microRNAs with histological subtypes, genetic driver alterations, therapeutically-targeted molecules, and carcinogens. The reported circulating microRNA signature for the early detection of lung cancer and the implications of microRNAs as the modulators of tumor immune response are also introduced.

## 1. Introduction

Lung cancer is the leading cause of cancer-related deaths in both men and women in developed as well as developing countries, accounting for more than 1.5 million deaths/year [1]. The two main histological lung cancer groups are small cell lung cancer (SCLC, 15% of all lung cancers) and non-SCLC (NSCLC, 85%); NSCLCs are further subclassified into adenocarcinoma, squamous cell carcinoma (SqCC), and large cell carcinoma. Accumulating lines of evidence suggest that lung cancer represents a group of histologically and molecularly heterogeneous diseases even within the same histological type [2,3,4,5,6]. Although progress has been recently made in the development of molecular-targeted drugs [7,8,9,10,11,12,13,14], potential targeted therapies can be administered to a limited number of lung cancer patients, not to all of them. Despite improvements in early detection of lung cancer, most lung cancers are diagnosed at an advanced stage. Thus, identifying novel diagnostic biomarkers and treatment strategies is critical and a prerequisite for managing lung cancer.

MicroRNAs are a class of small, single-stranded, evolutionarily conserved non-coding RNAs (19–22 nucleotides in length) that act as master regulators of gene expression, modulate almost all biological processes, and are essential for maintaining cellular homeostasis. They regulate gene expression at the post-transcriptional and translational levels by targeting the 3′-untranslated region (UTR) of messenger RNAs (mRNAs) [15]. Dysregulation of microRNA expression is often associated with the onset and progression of diseases or malignancies, including lung cancer [16,17,18,19,20,21,22,23,24,25,26,27]. Numerous microRNAs play important roles in lung cancer pathogenesis and have the potential to be diagnostic markers and therapeutically-targeted molecules. Thus, investigating the functional role of microRNAs will lead to a better understanding of lung carcinogenesis and open the door for effective diagnostic and therapeutic strategies to better manage lung cancers. Because many studies on lung cancer and microRNAs have been published, it is difficult to distinguish which microRNAs are actually associated with lung cancer or its clinicopathological features. Therefore, the study designs, including the sample sizes of the test and validation sets and in vitro/in vivo mechanistic studies, were the focus to maintain credibility.

In this review, recent studies of microRNAs in lung cancer are summarized, focusing on microRNAs as diagnostic and therapeutic tools.

## 2. MicroRNA Biogenesis

The biogenesis of microRNAs involves a complex process including multistep stages, as described previously [18]. MicroRNAs are initially transcribed by RNA polymerase II as primary-microRNAs with a hairpin structure. The DROSHA–DGCR8 enzyme complex then cleaves primary-microRNAs into precursor-microRNAs, which are transported to the cytoplasm by Exportin-5 (XPO5) and cleaved by DICER to yield microRNA duplexes. One strand is picked to function as a mature microRNA and loaded into the RNA-induced silencing complex (RISC), whereas the partner microRNA* is conversely degraded. The “*” notation indicates the passenger strand of the duplex, which could also function as listed in Table 1. The mature microRNAs lead to translational repression or degradation of target mRNAs.

## 3. MicroRNAs as Diagnostic Biomarkers

MicroRNAs are degraded to a much lesser extent in formalin-fixed paraffin-embedded (FFPE) samples than mRNAs, which are prone to degradation. Therefore, microRNAs can be accurately measured in FFPE specimens, which are usually collected and stocked in hospitals. The availability of archived FFPE specimens to accurately measure microRNAs allows us to perform translational studies using microRNAs.

An abundance of microRNAs exists not only in tissues but also in body fluids, such as blood and sputum [35], contrary to mRNAs. This property of easy availability makes microRNAs promising biomarkers in non-invasive liquid biopsies for cancer screening. Although, non-invasive liquid biopsies are promising [36,37,38,39], they are not used in cancer diagnosis due to the non-specificity of the body fluid-based microRNAs in identifying the primary cancer.

## 4. MicroRNAs and Histological Subtypes

The histological subclassification of lung cancers was modified by the 2015 WHO classification [2]. The major changes were as follows: (i) Adenocarcinoma was subclassified according to the invasive level from adenocarcinoma in situ (AIS), minimally invasive adenocarcinoma (MIA; invasive size ≤5 mm), and invasive adenocarcinoma (invasive size >5 mm). The terminology bronchioloalveolar carcinoma (BAC) was replaced by AIS; (ii) The terminology mixed adenocarcinoma was discontinued, and invasive adenocarcinoma was subclassified into one of the following five predominant patterns: lepidic, papillary, acinar, solid, or micropapillary. Variants of adenocarcinoma were redefined as invasive mucinous adenocarcinoma, colloid adenocarcinoma, fetal adenocarcinoma, and enteric adenocarcinoma; (iii) Undifferentiated carcinomas formerly classified as large cell carcinomas, expressing pneumocyte markers (TTF1 and/or Napsin A) or squamous differentiation markers (p40 and/or CK5/6) on immunohistochemistry were classified as adenocarcinoma (solid adenocarcinoma) or SqCC (non-keratinizing SqCC), respectively; (iv) A new category of “neuroendocrine tumors”, which include SCLC, large cell neuroendocrine carcinoma (LCNEC), and carcinoid tumor (typical and atypical), was established.

### 4.1. Adenocarcinoma

Nadal et al. conducted microRNA profiling of different subtypes of lung adenocarcinoma, and found that different morphological subtypes of lung adenocarcinoma have distinct microRNA expression profiles [33]. Hierarchical clustering of microRNAs divided lung adenocarcinomas into three clusters. MicroRNA clusters were highly associated with the predominant histological pattern based on the 2015 WHO classification [2]. Cluster 1 included fewer acinar and solid adenocarcinomas, whereas nearly all tumors were categorized as lepidic or invasive mucinous adenocarcinomas. On the other hand, clusters 2 and 3 were enriched in acinar and solid adenocarcinomas and included fewer lepidic and invasive mucinous adenocarcinomas. The top three microRNAs that were significantly associated with solid adenocarcinoma were *miR-27a*, *miR-212*, and *miR-132* (upregulation) [33].

Enteric adenocarcinoma was newly introduced as a variant of adenocarcinoma in the 2015 WHO classification [2]. Enteric adenocarcinoma is defined as an adenocarcinoma with a predominant enteric differentiation component [40]. Garajová et al. [41] examined microRNAs from enteric adenocarcinoma and reported that microRNA profiling of enteric adenocarcinoma reveals similarities with NSCLC and some overlap with pancreatic ductal adenocarcinoma (PDAC), but not with colorectal cancer. Enteric adenocarcinomas share key PDAC-associated microRNAs associated with tumor aggressiveness (miR-31*, miR-126*, *miR-506*, *miR-508-3p*, and *miR-514*). These findings could explain the aggressive behavior of lung enteric adenocarcinoma, thereby guiding future tailored-therapeutic approaches [41].

### 4.2. Squamous Cell Carcinoma (SqCC)

An appropriate histological subtyping is required to avoid hazardous side effects from new drugs for NSCLC. Bevacizumab, a monoclonal antibody that blocks angiogenesis by inhibiting vascular endothelial growth factor A (VEGFA), is not a suitable drug for patients with SqCC due to serious hemorrhagic complications. Similarly, pemetrexed, a chemotherapy drug in the folate antimetabolite class, is not suitable for patients with SqCC due to adverse responses. Several studies have distinguished SqCC from non-SqCC NSCLC using microRNA profiling [42,43,44]. Labanony et al. reported that high expression of *miR-205* is specific to SqCC, and *miR-205* expression distinguishes SqCCs from non-SqCC NSCLCs [42]. Bishop et al. showed that *miR-205* expression in small biopsies or aspirates can distinguish SqCCs from non-SqCC NSCLCs [43]. Hashimoto et al. reported that the expression of three microRNAs (*miR-205*, *miR-196b*, and *miR-375*) can be used to distinguish SqCCs from non-SqCC NSCLCs [44].

### 4.3. Small Cell Lung Carcinoma (SCLC)

SCLC, which is a neuroendocrine tumor, exhibits increased expression of ASCL1, which is a transcription factor that promotes neuroendocrine differentiation. Nishikawa et al. reported that *miR-375* expression was promoted by ASCL1 in lung neuroendocrine carcinoma [45]. They suggested that the *miR-375* might reduce the YAP1-related proliferative arrest by inhibiting YAP1. Yu et al. examined microRNAs from 50 SCLC patients and 30 healthy controls, and suggested that *miR-92a-2* level in plasma could be a potential and non-invasive method for the diagnosis of SCLC [46]. Recently, SCLC and large cell neuroendocrine carcinoma (LCNEC) have been classified into the same category of “neuroendocrine tumors” according to the 2015 WHO classification [2]. Demes et al. examined the association of *miR-21* and *miR-34a* expression in neuroendocrine tumors, and found that *miR-21* expression was higher in high-grade neuroendocrine tumor (HGNET; i.e., SCLC and LCNEC) than in typical/atypical carcinoid, and high-expression level of *miR-34a* was associated with atypical carcinoids [47].

## 5. MicroRNAs and Genetic Drivers

Somatic genetic alterations in tyrosine kinase have emerged as driver genetic alterations in lung carcinogenesis, especially in lung adenocarcinoma [12]. Driver genetic alterations in lung adenocarcinoma include rearrangements of *ALK*, *RET*, *ROS1*, *NTKR1*, *NRG2*, *ERBB4*, and *BRAF* and mutations of *EGFR*, *KRAS*, *BRAF*, *ERBB2*, *NRAS*, *HRAS*, *MAP2K1*, *NF1*, and *RIT1* [3,48,49,50,51,52]. Adenocarcinoma with specific genetic alterations sometimes have characteristic clinicopathological features [53,54,55,56,57].

Several studies examined the association between microRNAs expression and driver genetic alterations. Bjaanaes et al. examined microRNA profiles according to mutation status of *EGFR* and *KRAS* [28]. They identified 17 microRNAs that were differentially expressed between *EGFR*-mutated and *EGFR*-wild-type lung adenocarcinomas, and 3 microRNAs differentially expressed between *KRAS*-mutated and *KRAS*-wild-type lung adenocarcinomas (Table 1). Gasparini et al. assessed microRNA profiles of NSCLCs driven by rearranged *ALK*, mutated *EGFR*, or mutated *KRAS* to find driver specific microRNA signatures [58]. They identified that expression levels of *miR-1253*, *miR-504*, and *miR-26a-5p* could classify NSCLCs as rearranged-*ALK*, mutated *EGFR*, or mutated *KRAS* versus wild type. Recently, Kim et al. examined microRNA expression profiles according to *EGFR*, *KRAS*, and *ALK* status [29]. They found that five microRNAs (Overexpression of *miR-1343-3p* and Underexpression of *miR-671-3p*, *miR-103a-3p*, *let-7e*, and *miR-342-3p*) were especially distinctive in the *ALK*-rearranged group, compared to *EGFR*-mutated and *KRAS*-mutated groups (Table 1). For now, limited evidence is available for the microRNA profiles of lung cancer with specific driver genetic alterations. Further studies are warranted to investigate them for the combination therapy of tyrosine kinase inhibitors with microRNA-based treatments.

## 6. MicroRNAs and Therapeutically-Targeted Molecules

Aside from targeting driver mutations, emerging evidence suggests that the other molecular targets are promising for the treatment of lung cancer.

### 6.1. PD-L1

The immune checkpoints mechanism plays a key role in suppressing the anti-tumor T-cell-mediated immune response in the tumor microenvironment. PD-L1 (also known as CD274 and B7-H1) is an immune modulator that promotes immunosuppression by binding to PD-1 (also known as PDCD1) of T-lymphocytes. Therapeutic antibodies targeting PD-1 and PD-L1 have been shown to be effective in many cancer types, including lung cancer (both NSCLC and SCLC). PD-L1 expression in cancer cells has been suggested as a predictive marker of the clinical response to PD-1/PD-L1-targeted therapy [10,59,60]. PD-L1 positivity in lung adenocarcinoma has been associated with higher mortality and *EGFR* wild-type status [61].

PD-L1 has been reported to be regulated by TP53 via *miR-34* [30]. The 3′-UTR of the *PD-L1* mRNA carries a putative *miR-34* binding site. In models of NSCLC, TP53 transcriptionally promoted *miR-34* expression, and increased *miR-34* targeted *PD-L1* mRNA, leading to the decrease of PD-L1 protein (Table 1) [30]. Because they demonstrated that the therapeutic delivery of *miR-34a* combined with standard therapies, such as radiotherapy, demonstrated a better clinical response, this combined therapy may represent a new mode of immunotherapy [30].

### 6.2. B7-H3

B7-H3 (also known as CD276) belongs to a family of immune modulators (known as the B7 family) that includes PD-L1 (or B7-H1), and has been associated tumor immunosuppression and decreased survival of cancer patients [62]. Targeting B7-H3 is a potential treatment of cancer [63]. In lung adenocarcinoma, higher expression of B7-H3 has been associated with *EGFR* wild-type and smoking patients [64].

*MiR-29a* was shown to directly target the 3′-UTR of *B7-H3* mRNA (Table 1) [31]. Knock-in and knockdown of *miR-29a* led to downregulation and upregulation, respectively, of B7-H3 protein expression in cell lines [31]. Therefore, therapeutic delivery of *miR-29a* may be effective for the treatment for cancer with high expression of B7-H3. As mentioned below, B7-H3 protein reduces the anti-tumor activity mediated by T-lymphocytes and NK cells by sending an inhibitory signal to them. Therefore, *miR-29a* plays a role as the modulator of tumor immune response.

### 6.3. TROP2

TROP2 (also known as TACSTD2) is a transmembrane glycoprotein that is highly expressed in many cancers, and is a promising molecular target for the treatment of various malignancies [65]. Sacituzumab govitecan (IMMU-132) is an anti-TROP2 antibody-drug conjugate that contains SN-38, the active metabolite of irinotecan. Without severe side effects, IMMU-132 has been effective against metastatic SCLC [66] and metastatic NSCLC resistant to anti-PD-1/PD-L1 therapy [67]. Association of tumor TROP2 expression with prognosis has been shown to vary among lung cancer subtypes. High TROP2 expression is associated with high mortality in adenocarcinoma and low mortality in HGNET but is not associated with mortality in SqCC [68].

TROP2 was identified as a direct and functional target of *miR-125b-1* in cell lines (Table 1) [32]. Loss of *miR-125b-1* was reported to promote head and neck carcinogenesis by dysregulating TROP2 and MAPK pathway [32]. The association between *miR-125b-1* and TROP2 expression warrants investigation in lung cancer. *MiR-125b-1* may directly downregulate TROP2 expression and contribute to the activation of the MAPK pathway in lung cancer. This pathway can be targeted by drugs, providing a novel therapeutic opportunity for lung cancer.

## 7. MicroRNAs and Carcinogens

Cigarette smoke and asbestos are major carcinogens of lung cancer. Smoking cigarettes, containing mutagens, is a primary risk factor for the development of lung cancer. The carcinogens in cigarette smoke are distinct from asbestos, and the major carcinogenic mechanism of asbestos was suggested to be tumor promotion, acting in an additive or synergistic manner, contributing to the genotoxic effect of smoking [69].

### 7.1. Cigarette Smoke

Nodal et al. conducted microRNAs profiling of lung adenocarcinoma, and found that *miR-210* was positively associated with pack-years consumed (Table 1). On the other hand, *miR-342*, *miR-151*, *miR-501-3p*, *miR-29b*, *miR-30d*, *miR-497*, *miR-222*, *miR-505*, *miR-34b*, *miR-500*, and *miR-99a-3p* were inversely correlated with pack-years consumed (Table 1) [33]. *MiR-210*, which was highly expressed in heavy-smoking patients’ lung adenocarcinoma, has been associated with hypoxia in lung cancer and positively regulates HIF-1a [70]. Certain microRNAs negatively correlated with pack-years may act as tumor suppressor microRNAs, and their expression may be regulated by epigenetic mechanisms [71]. Lu et al. found that lncRNA *CCAT1* and c-Myc might be involved in cigarette smoke extract-induced lung carcinogenesis via *let-7c*. Feedback circuitry via *let-7c* between *CCAT1* and c-Myc might be involved in smoking-associated lung cancers [72].

### 7.2. Asbestos

Nymark et al. conducted an integrative analysis using lung cancer tissues and corresponding normal lung tissues from asbestos-exposed and non-exposed patients. They identified asbestos-related microRNA (Overexpression of *miR-148b*, *miR-374a*, miR-24-1*, *let-7d*, *let-7e*, *miR-199b-5p*, *miR-331-3p*, and *miR-96*; Underexpression of *miR-939*, *miR-671-5p*, *miR-605*, *miR-1224-5p*, and *miR-202*) (Table 1) and inversely correlated target genes (e.g., *GADD45A*, *LTBP1*, *FOSB*, *NCALD*, *CACNA2D2*, *MTSS1*, and *EPB41L3*) [34].

## 8. Circulating MicroRNAs Signature for Early Detection of Lung Cancer

Importantly, microRNAs are present in stable form not only in tissues but also in body fluids (e.g., blood, plasma, serum, or sputum). Several studies have demonstrated that the plasma or serum microRNA (i.e., circulating microRNAs) signature has a potential clinical value in the early detection of lung cancer and would play a critical role in the preliminary screening of lung cancer in the general population [73,74,75,76,77,78,79,80,81,82]. Geng et al. examined 25 early-stage NSCLC patients and 25 matched healthy controls for the training set and 126 early-stage NSCLC patients, 42 non-cancerous pulmonary disease patients, and 60 healthy controls for the validation set. They demonstrated that five plasma microRNAs (*miR-20a*, *miR-145*, *miR-21*, *miR-223*, and *miR-221*) could be used as promising biomarkers for the early screening of NSCLC [73]. Using a large prospective early detection trial (the COSMOS study) for lung cancer by low-dose computed tomography, Bianchi et al. investigated serum microRNAs and identified a 34-microRNA signature that could identify patients with early-stage NSCLC in a population of asymptomatic high-risk individuals with 80% accuracy [74]. They further ameliorated the signature of microRNAs by decreasing the involved microRNAs to a 13-microRNA signature (*miR-92a-3p*, *miR-30b-5p*, *miR-191-5p*, *miR-484*, *miR-328-3p*, *miR-30c-5p*, *miR-374a-5p*, *let-7d-5p*, *miR-331-3p*, *miR-29a-3p*, *miR-148a-3p*, *miR-223-3p*, and *miR-140-5p*) and conducted a validation study using a larger cohort. The validation study conducted on 1,115 independent high-risk individuals enrolled in the COSMOS study exhibited 75% accuracy, 78% sensitivity, and 75% specificity [75]. Nadal et al. examined 70 NSCLC patients and 22 controls for serum microRNAs and identified a four-microRNA signature (*miR-193b*, *miR-301*, *miR-141*, and *miR-200b*) that could differentiate NSCLC patients from non-cancer individuals. In the validation study, the four-microRNA signature yielded 97% sensitivity and 96% specificity in distinguishing 84 NSCLC patients from 23 non-cancer individuals [77]. Studies on circulating microRNA signature for the early detection of lung cancer share a few microRNAs. Further studies with a large sample size are required to apply the circulating microRNA signature to distinguish lung cancer patients from non-cancer individuals.

## 9. Implications of MicroRNAs as Modulators of Tumor Immune Response

MicroRNAs play important roles in the negative regulation of immune responses in many cancer cells [83]. The attenuated recognition of cancer cells by immune cells can be caused by alterations in antigenic patterns because of the genetic instability of cancer cells. A number of microRNAs contribute to these antigenic alterations. *MiR-9*, which is overexpressed in several malignancies, including lung cancer [84], downregulates MHC class I, thereby preventing the detection of cancer cells by the immune system [85]. *MiR-222* and *miR-339* downregulate ICAM1 expression on cancer cell membranes [86]. Because ICAM1 is essential for activating cytotoxic T-lymphocytes (CTLs), *miR-222* and *miR-339* in tumor cells downregulate the susceptibility of tumor cells to CTL-mediated cytolysis [86]. *MiR-29* targets and downregulates B7-H3 (CD276). Therefore, *miR-29* downregulation in tumor cells leads to B7-H3 upregulation, which promotes B7-H3 protein expression in tumor cell membranes [31]. As a result, B7-H3 sends an inhibitory signal to T-lymphocytes and NK cells, thereby reducing the anti-tumor activity mediated by them. As mentioned above, microRNAs play several roles as the modulators of the tumor immune response. Therefore, microRNAs can be potential immunotherapeutic agents.

## 10. Conclusions

In this review, recent works of microRNAs were introduced, with a particular interest in microRNAs associated with histological subtypes, genetic driver alterations, therapeutically-targeted molecules, and carcinogens in lung cancer. The circulating microRNA signature for the early detection of lung cancer and the implication of microRNAs as the modulators of tumor immune response are also introduced. Because microRNAs are far less degradated in FFPE samples than mRNAs and are present not only in cancer tissues, but also in body fluids, there is an increased potential in microRNA analyses for cancer research. Further research is warranted to use microRNAs as diagnostic markers and conduct microRNA-based treatments in clinical practice.

## Figures and Tables

**Table 1:** Association of microRNAs with genetic drivers, therapeutically-targeted molecules, and carcinogens.

Various Features	MicroRNA
Genetic drivers	
*EGFR* mutation	Overexpression: *miR-184*, *miR-339-3p*, miR-148a*, miR-224*, *miR-452*, *miR-450a*, *miR-423-3p*, *miR-654-5p*, *miR-532-5p*, *miR-3607-5p*, *miR-28-3p*, miR-30d*, *miR-532-3p*, miR-500a*, *miR-502-3p*, *miR-605* [28] Underexpression: *miR-492* [28]
*KRAS* mutation	Overexpression: *miR-100* [28] Underexpression: *miR-371-5p*, *miR-564* [28]
*ALK* rearrangement	Overexpression: *miR-1343-3p* [29] Underexpression: *miR-671-3p*, *miR-103a-3p*, *let-7e*, *miR-342-3p* [29]
Therapeutically-targeted molecules	
PD-L1 (CD274)	*MiR-34* targets PD-L1 [30]
B7-H3 (CD276)	*MiR-29a* targets B7-H3 [31]
TROP2 (TACSTD2)	*MiR-125b-1* targets TROP2 [32]
Carcinogens	
Cigarette smoke	Overexpression: *miR-210* [33] Underexpression: *miR-342*, *miR-151*, *miR-501-3p*, *miR-29b*, *miR-30d*, *miR-497*, *miR-222*, *miR-505*, *miR-34b*, *miR-500*, *miR-99a-3p* [33]
Asbestos	Overexpression: *miR-148b*, *miR-374a*, miR-24-1*, *let-7d*, *let-7e*, *miR-199b-5p*, *miR-331-3p*, *miR-96* [34] Underexpression: *miR-939*, *miR-671-5p*, *miR-605*, *miR-1224-5p*, *miR-202* [34]

In microRNA biogenesis, one strand of microRNA duplexes is picked to function as a mature microRNA and loaded into the RNA-induced silencing complex (RISC), whereas the partner microRNA* is conversely degraded. The “*” notation indicates the passenger strand of the duplex, which could also function as listed.

## Abbreviations

AIS	adenocarcinoma in situ
BAC	bronchioloalveolar carcinoma
CTL	cytotoxic T-lymphocyte
FFPE	formalin-fixed paraffin-embedded
HGNET	high-grade neuroendocrine tumor
LCNEC	large cell neuroendocrine carcinoma
MIA	minimally invasive adenocarcinoma
mRNA	messenger RNA
NSCLC	non-small cell lung cancer
PDAC	pancreatic ductal adenocarcinoma
RISC	RNA-induced silencing complex
SCLC	small cell lung cancer
SqCC	squamous cell carcinoma
UTR	untranslated region
VEGFA	vascular endothelial growth factor A
